# The val^158^met polymorphism of human catechol-O-methyltransferase (*COMT*) affects anterior cingulate cortex activation in response to painful laser stimulation

**DOI:** 10.1186/1744-8069-6-32

**Published:** 2010-05-31

**Authors:** Arian Mobascher, Juergen Brinkmeyer, Holger Thiele, Mohammad R Toliat, Michael Steffens, Tracy Warbrick, Francesco Musso, Hans-Joerg Wittsack, Andreas Saleh, Alfons Schnitzler, Georg Winterer

**Affiliations:** 1 Department of Psychiatry, Johannes Gutenberg-University, Untere Zahlbacher Str. 8, 55131, Germany; 2 Department of Psychiatry, Heinrich-Heine University, Bergische Landstr. 2, 40629 Duesseldorf, Germany; 3 Institute of Neurosciences and Biophysics, Research Center Juelich, 52425 Juelich, Germany; 4 Cologne Center for Genomics, Institute for Genetics, University of Cologne, Weyertal 115b, 50931 Köln, Germany; 5 Institute of Medical Biometry, Informatics and Epidemiology, University of Bonn, Sigmund-Freud-Str. 25, 53105 Bonn, Germany; 6 Institute of Radiology, Heinrich-Heine University, Moorenstr. 5, 40225 Duesseldorf, Germany; 7 Insitute of Clinical Neurosciences and Medical Psychology, Heinrich-Heine University, Universitätsstr. 1, 40225 Duesseldorf, Germany

## Abstract

**Background:**

Pain is a complex experience with sensory, emotional and cognitive aspects. Genetic and environmental factors contribute to pain-related phenotypes such as chronic pain states. Genetic variations in the gene coding for catechol-O-methyltransferase (*COMT*) have been suggested to affect clinical and experimental pain-related phenotypes including regional μ-opioid system responses to painful stimulation as measured by ligand-PET (positron emission tomography). The functional val^158^met single nucleotide polymorphism has been most widely studied. However, apart from its impact on pain-induced opioid release the effect of this genetic variation on cerebral pain processing has not been studied with activation measures such as functional magnetic resonance imaging (fMRI), PET or electroencephalography. In the present fMRI study we therefore sought to investigate the impact of the *COMT *val^158^met polymorphism on the blood oxygen level-dependent (BOLD) response to painful laser stimulation.

**Results:**

57 subjects were studied. We found that subjects homozygous for the met158 allele exhibit a higher BOLD response in the anterior cingulate cortex (ACC), foremost in the mid-cingulate cortex, than carriers of the val158 allele.

**Conclusion:**

This result is in line with previous studies that reported higher pain sensitivity in homozygous met carriers. It adds to the current literature in suggesting that this behavioral phenotype may be mediated by, or is at least associated with, increased ACC activity. More generally, apart from one report that focused on pain-induced opioid release, this is the first functional neuroimaging study showing an effect of the *COMT *val^158^met polymorphism on cerebral pain processing.

## Background

Pain is a multidimensional construct embodying sensory, affective and cognitive components [1] exhibiting a high degree of inter-individual variability in clinical and experimental settings [2,3]. Twin studies suggest that genetic factors contribute to the observed inter-individual differences in pain-related phenotypes with heritability estimates of up to 70% for clinical pain conditions [2,3] and up to 60% for sensitivity to certain kinds of experimental stimuli [4]. In view of the complexity of pain processing with regard to the neuroanatomical structures/networks and neurochemical systems involved [5,6], single nucleotide polymorphisms (SNPs) in multiple genes can be expected to contribute to the overall heritability of pain-related phenotypes.

Brain activity as measured by functional neuroimaging has been shown to correlate with subjective pain experience [7-9]. Therefore brain activation measures may serve as intermediate phenotypes when genetic aspects of pain behavior are studied. However, only a few studies have applied functional magnetic resonance imaging (fMRI) or positron emission tomography (PET) to investigate the impact of inter-individual genetic differences on cerebral pain processing [10,11]. One of the few SNPs that have been studied with regard to both behavioral pain measures and brain activity as measured by neuroimaging is a common functional variant in the gene coding for catechol-O-methyltransferase (*COMT*). *COMT *is an enzyme that controls the breakdown of catecholamines in the brain. The *COMT *val^158^met SNP (rs4680) - in which valine is replaced by methionine at position 158 of the amino acid chain - has been shown to affect opiate requirements in clinical settings and sensitivity to experimental pain [10,12,13]. Using μ-opoid receptor ligand-PET, Zubieta et al. also showed that this SNP affects the activity of the endogenous opioid system upon painful stimulation. Subjects homozygous for the met158 allele have been reported to exhibit highest pain sensitivity with subjects homozygous for the val158 allele showing the opposite phenotype. On the other hand, there are also several studies that reported no effect of this SNP on pain experience [14,15].

Intermediate phenotypes as revealed by neuroimaging techniques such as fMRI are thought to be more closely linked to the genetics of a complex behavior and neuropsychiatric disorders than the behavior/the disorder itself [16]. To our knowledge no imaging studies on the impact of the COMT val^158^met polymorphism on brain activity upon painful stimulation have been published - apart from the molecular imaging study of Zubieta and colleagues [10] using μ-opoid receptor ligand-PET. Therefore, functional studies to assess the impact of the COMT genotype on cerebral pain processing are warranted. In the present study, the COMT val^158^met genotype effect on the fMRI BOLD (blood-oxygen-level-dependent) response to painful laser stimulation was investigated.

## Results and Discussion

### BOLD response to laser stimulation

**Figure 1:**
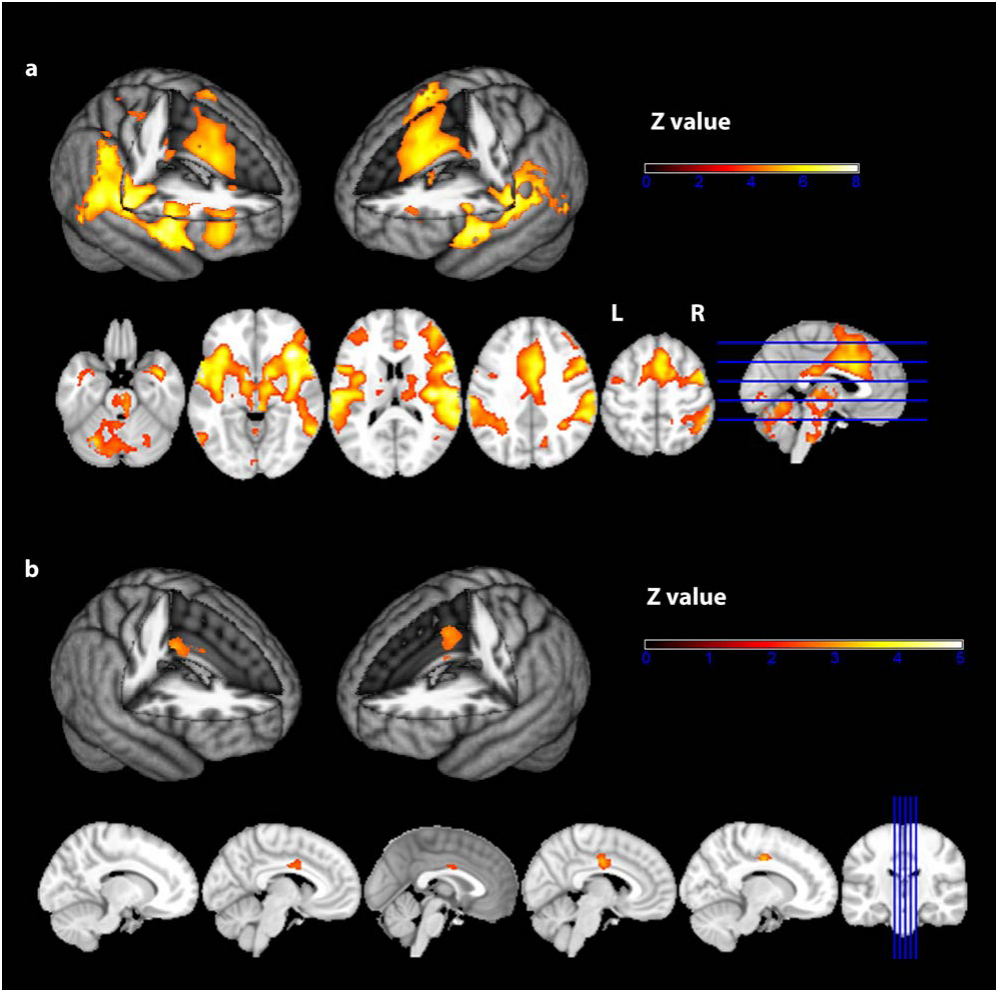
**BOLD response to laser stimulation**. a) Group average. N = 57 subjects. GLM whole-brain analysis. Second-level mixed-effects FLAME. Cluster-corrected threshold Z = 3.5, p = 0.05. Upper row: 3 D surface projection. Lower row: axial slices. R = right. L = left. b) Two-group t-test. *COMT*met/met (N = 19) vs. *COMT*val carriers (N = 38). Voxel-by-voxel analysis restricted to the anterior cingulate cortex (according to the Harvard Oxford atlas). Second-level mixed-effects FLAME. Cluster-corrected threshold Z = 2.3, p = 0.05. Upper row: 3 D surface projection. Lower row: sagittal slices.

Voxelwise analysis revealed significant BOLD responses to laser stimuli in the entire pain matrix including cortical and subcortical areas such as contralateral primary somatosensory cortex (S1), bilateral secondary somatosensory cortex (S2), bilateral insula, anterior cingulate cortex (ACC), precuneus, cerebellum thalamus and brainstem (see Fig. 1 and Table 1 for detailed information).

**Table 1:** BOLD activation in response to laser stimulation

Region	MNI coordinates (X Y Z)	Peak activation (Z)
R. parietal operculum	62 -24 20	7.68

R. insula	36 24 -4	8.13

R. postcentral gyrus	42 -36 58	3.80

R. amygdala	22 0 -16	5.25

R. precentral gyrus	44 -2 58	6.34

R. (pre)frontal cortex	46 38 2	5.84

L. parietal operculum	-58 -24 18	7.12

L. insula	-36 20 2	7.94

L. (pre)frontal cortex	-38 44 20	4.60

L. amygdala	-24 0 -18	4.46

ACC/mid-cingulate	6 12 34	6.56

Precuneus	12 -70 40	5.28

cerebellum	-30 -60 -34	5.80

R. thalamus	14 -10 2	6.26

L. thalamus	12 -12 -2	5.23

Midbrain	10 -24 -14	5.95

Brainstem	2 -30 -46	4.92

R = right, L = left

### Factors determining BOLD activation

**Figure 2:**
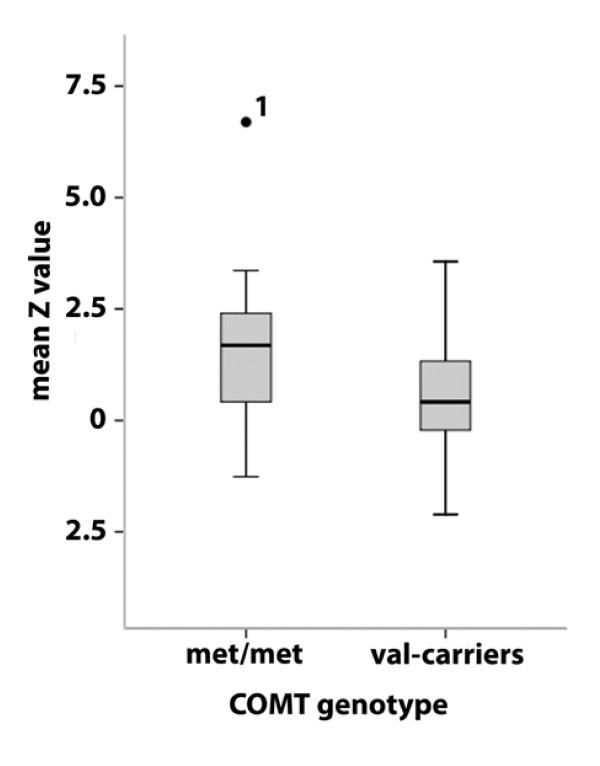
**Boxplot of *COMT *genotype effect on fMRI BOLD activation in the anterior cingulate cortex (ACC)**. Median, 25^th ^percentile, 75^th ^percentile, minimum value, maximum value and outliers are shown. ^1 ^The high BOLD activation in response to laser stimulation in this subject was reproduced in a second experiment that was performed two month later (mean Z value in the ACC = 5.3), suggesting that this 'outlier' is reflecting biological variance in the sample and not a measurement error. Furthermore, the genotype effect on ACC activation remained significant, (F = 7.06; p = 0.01; F = 4.92; p = 0.031 age-corrected) even when this subject was excluded from the analysis.

Exploratory region-of-interest (ROI) analyses revealed no significant effects of gender or smoking status on BOLD activation (mean Z values) in the parietal opercular cortex, the insula, the ACC or the amygdalae (data not shown). However age was negatively correlated with mean Z values in all four ROIs (Pearson correlation coefficients: r = -0.401 (ACC), r = -0.450 (S2), r = -0.481 (insula) and r = -0.402 (amygdalae); p < 0.002). The general linear model (GLM) multivariate analysis of variance (MANOVA) revealed a statistically significant *COMT *genotype effect on BOLD activation across the four ROIs (F = 2.794, df = 4, p = 0.035). Subsequent analyses of variance (ANOVAs) four each ROI showed a significant genotype effect in three out of four ROIs (trend finding in the bilateral amygdala) with subjects homozygous for the met158 allele consistently exhibiting higher mean BOLD activation (see Table 2 for detailed information). When the ANOVAs were corrected for age, only the genotype effect on BOLD activation in the ACC remained statistically significant (F = 6.32; p = 0.015; df = 1). In Fig. 2 the effect of *COMT *genotype on ACC activation is shown. Similar results were obtained, when only the population-based subsample of N = 47 subjects was analysed (data not shown).

**Table 2:** Impact of COMT genotype on fMRI BOLD activation

Region of Interest	Genotype (df = 1)			
	**Met/Met** **(Mean Z value/SD)**	**Val carriers** **(Mean Z value/SD)**	**F**	**p**

ACC	1.69/1.75	0.55/1.05	9.447	0.003

Insula	2.29/1.88	1.40/1.08	5.136	0.027

parietal operculum (containing S2)	2.13/1.75	1.36/0.94	4.677	0.035

Amygdalae	1.09/1.48	0.52/0.85	3.507	0.066

Analyses of variance (ANOVAs) with genotype as factor and mean Z values in regions of interest as dependent variables.

df = degrees of freedom; ACC = anterior cingulate cortex; S2 = secondary somatosensory cortex; SD = standard deviation

As the ACC is a large structure in which subregions serve distinct purposes in cortical pain processing, we next sought to investigate which subregion(s) of the ACC were affected by *COMT *genotype with respect to laser-induced BOLD activation. The voxel-by-voxel analysis restricted to the ACC showed that subjects homozygous for the met158 allele exhibited higher BOLD activation in the posterior ACC/mid-cingulate cortex (Z_max _= 3.37; MNI coordinates x/y/z: 10/-10/38). See also Fig. 1b.

No correlation between post-hoc pain ratings and BOLD activation (as measured by mean Z values in the ROIs) was observed (Pearson correlation coefficients: r = 0.162 (ACC), r = 0.054 (S2), r = 0.096 (insula) and r = 0.181 (amygdalae); p > 0.177). Likewise, no statistically significant effect of genotype on post-hoc pain ratings was found (met/met genotype: mean 41.4/SD 14.2; val carriers: mean 42.4/SD 16.4; F = 0.05; p = 0.824, df = 1).

## Discussion

In the present study we sought to investigate the impact of the *COMT *val^158^met single nucleotide polymorphism on cerebral pain processing as measured by fMRI.

The overall BOLD activation pattern in response to painful laser stimulation in our study is consistent with numerous previous imaging studies showing activation of a distributed network of cortical and subcortical structures including the core regions of cerebral pain processing (often referred to as the 'pain matrix') such as the bilateral parietal operculum containing the secondary somato-sensory cortex (S2), bilateral insula and bilateral anterior cingulate cortex (ACC)/mid-cingulate cortex (MCC) [5,6,17].

Subjects homozygous for the met158 allele exhibited a higher BOLD response to laser stimulation than carriers of the val158 allele (homozygous and heterozygous val158 carriers combined) in key areas of the 'pain matrix' - i.e. in S2, the insula the amygdalae and foremost in the ACC. In the voxel-by-voxel analysis restricted to the ACC we found the most significant activation difference between genotype groups in the posterior portion of the ACC/the MCC.

The ACC has been shown to be a key structure of cortical pain processing that is involved in the cognitive/emotional evaluation of pain as well as in antinociception [5,6,18-21]. Furthermore, Büchel et al. [8] showed that activation in the posterior ACC/MCC is correlated with stimulus intensity and pain ratings. Therefore our data are consistent with previous studies that found an association of the *COMT *met158 allele with higher pain ratings [10,13] and with altered μ-opioid receptor density and opioid responses to pain in several brain areas including the dorsal ACC [10]. Our results add to the current literature in that they suggest that COMT-dependent ACC/MCC activity plays a prominent role in mediating sensitivity to pain, possibly due to a reduction of opioid-mediated inhibitory control. However, it needs to be pointed out that negative findings with regard to the effect of this SNP on pain-related phenotypes have also been reported [14,15]. This discrepancy may be explained by sample heterogeneity, differences in the modality used to elicit pain and by differences in the behavioral readout. Furthermore it has been suggested that haplotypes that also include other SNPs in *COMT *may exert a stronger effect on pain-related phenotypes [22,23] than the *COMT *val^158^met polymorphism alone. We observed an effect of COMT genotype on brain activation in the absence of any behavioral effects (subjective pain ratings). This discrepancy may be explained by different effect sizes of imaging endophenotypes and behavioral phenotypes with larger genotype effects on brain activation than on overt behavior [16,24].

The COMT val^158^met polymorphism may affect pain processing in several ways. The met158 allele codes for an enzyme variant that is less stable at body temperature leading to higher dopamine levels in the brain, mainly in - but not restricted to - the (pre)frontal cortex. Dopamine levels may modulate the activity of the endogenous opioid system indirectly by regulating the neuronal content of enkephalins [10,25,26]. This could affect activation measures in brain areas involved in processing and modulation of painful stimuli and ultimately behavioral pain-related phenotypes [10]. Alternatively/additionally, *COMT *may affect pain processing by decreasing the metabolism of epinephrine which has also been shown to modulate pain processing. For instance, Khasar et al. [27] showed that β_2_-adrenergic stimulation induces hyperalgesia in the rat. Furthermore it has been shown that a COMT inhibition-induced increase in pain sensitivity is blocked by beta-adrenergic antagonists [28]. Yet another alternative may be that the *COMT *val^158^met SNP affects brain activity in response to painful stimulation in a rather unspecific way: a substantial amount of genetic imaging papers found an impact of this SNP on brain activity in various cognitive domains such as attention, working memory and affective regulation [29]. The genotype effect on pain processing that we found in the present study may therefore be 'downstream' of a more direct influence on attention or affect regulation. For instance, Smolka et al. [30] showed that compared to carriers of the val allele subjects homozygous for the met allele exhibit an increased BOLD response to unpleasant pictures in a partially overlapping network of structures including the amygdala. Therefore homozygous met carriers may be more reactive to a variety of negative stimuli including pain. However, activation in the dorsal ACC/MCC - the site of the most significant genotype effect in our study and a key structure of the pain matrix - was not affected by *COMT *genotype in the study by Smolka et al. This region of the ACC has been implicated in several aspects of pain processing such as encoding pain- and stimulus intensity [8]. Furthermore the dorsal ACC has been shown to be one of the sites of pain-induced opioid release [19]. In a subsequent paper Zubieta et al. [10] showed that the *COMT *val^158^met polymorphism affects the μ-opioid response to painful stimulation in the dorsal ACC. Last, it has been shown that the ACC/MCC is the main generator of the N2 and P2 laser-evoked potentials in studies using EEG source localization [31], intracranial recordings [32] or more recently EEG-informed fMRI [33]. All this may suggest that the observed effect of *COMT *genotype on fMRI BOLD activation is not entirely unspecific. However, it has recently been shown that laser-evoked potentials themselves are not nociceptive-specific [34].

The genetic basis of pain perception and processing has mostly been studied in clinical settings such as chronic pain states, post-surgical pain or experimental settings in which tonic pain models were applied. Correspondingly, an effect of *COMT *genotype on pain-related phenotypes has for instance been shown in cancer patients with regard to analgesic requirements [12,35] and in experiments in which models of sustained pain such as the intramuscular injection of hypertonic saline [10]; tonic heat pain [13] or the temporal summation of thermal pain were studied [22]. By contrast laser stimulation of the skin as applied in our study induces phasic pain which is quite different from tonic pain models or clinical pain states. An effect of *COMT *genotype on phasic pain has not been reported so far. Therefore our findings require further replication and cannot readily be transferred to clinical pain states and their genetic basis.

Our study has several limitations. First, our sample comprised only 9 subjects with *COMT *val/val genotype, a number that we considered to low to constitute a separate group in our genetic fMRI analyses. Thus, we combined homozygous and heterozygous val158 carriers in one group which leaves the question of additive vs. dominant/recessive gene effects unanswered. Second, due to technical reasons we did not obtain continuous online pain ratings, but only post-hoc ratings which are considered less accurate. This could explain the lack of a relationship between pain ratings on one hand and *COMT *genotype and fMRI BOLD activation on the other hand. Then again genotype effects are well known to require comparatively large samples to be detected when behavioral measures and probably even more so subjective measures like pain ratings are used which is one of the main reasons why intermediate phenotypes like brain activation as measured by fMRI are studied [16]. Third, our paradigm did not include a control condition with innoxious stimuli. While laser stimulation of the skin provides Aδ and C fibre-mediated nociceptive-specific input to the brain, cerebral processing of that input has been shown not to be pain-specific [34]. Therefore we cannot rule out the possibility that the observed differences between *COMT *genotype groups in the BOLD activation pattern reflect a more general genotype effect on brain activity especially as *COMT *affects several aspects of human behavior including, attention working memory and emotional regulation [29]. Lastly, we investigated a rather heterogeneous sample, which is reflected by the high impact of age on BOLD activation measures in our study. Heterogeneity of the sample may increase the background variance in the data diminishing the effect of the genetic variant that is under investigation [2]. On the other hand, the genotype effect survived age correction and we found an impact of the *COMT*^158^val/met on ACC/MCC activation in a sample that was largely selected from a population-based sample even after age correction. The latter may in fact be considered a plus with respect to the generalizability of the results of a genetic imaging study.

## Conclusion

To our knowledge, this is the first fMRI study showing an effect of the *COMT *val^158^met polymorphism on brain activation - mainly in the posterior ACC - in response to experimental pain. We consider this an important independent verification of previous work using a μ-opioid specific PET ligand that suggested that this SNP affects the neurobiology of pain processing in related brain regions. More generally our study provides further evidence that pain-related intermediate phenotypes revealed by neuroimaging methods such as fMRI may be a useful concept to study genotype-phenotype relationships in pain research.

## Methods

### Subjects

A total of N = 57 healthy subjects (27 males) with a mean age of 35.3 (SD 11.1) years were recruited both from a larger population-based sample that will be described in more detail elsewhere (Mobascher et al., unpublished data, N = 47 subjects) and from the environment of the local university (students or staff, N = 10 subjects). Because of the small number of subjects homozygous for the *COMT *val158 allele (N = 9) - which may be considered too low for the purposes of genetic fMRI - this group was combined with the group of heterozygous *COMT *val/met carriers for subsequent analyses. Demographic data for both genotype groups (*COMT *met/met vs. *COMT *val carriers, i.e. *COMT*val/met + *COMT*val/val) are provided in Table 3. Subjects had no history of neurological or psychiatric disease and did not take any medication that could affect the experiment. To minimize potential confounding effects of the female hormonal cycle on pain processing [36,37] all female subjects were investigated during the follicular phase of the menstrual cycle. All subjects had normal pain thresholds (350-500 mJ) as determined prior to the imaging experiment using a series of laser stimuli increasing in steps of 50 mJ from 200 to 600 mJ. Subjects were asked to report the point at which the sensation could be described as painful. This procedure was repeated with decreasing steps of 50 mJ from 600 to 200 mJ with subjects reporting when the sensation was no longer painful. The mean of these two values was taken as pain threshold. Subjects gave written informed consent to participate in the study. The study was conducted in compliance with the declaration of Helsinki and was approved by the local ethics committee.

**Table 3:** Demographic data

	COMT Genotype group		Statistical significance of group differences
	
	met/met N = 19	val carriers N = 38	
Males/females	12/7	15/23	(χ^2 ^= 2.85; p = 0.091)

Smokers/Non-smokers	7/12	17/21	(χ^2 ^= 0.32; p = 0.569)

Mean age (years)/SD	31.6/11.0	37.2/10.9	(t = 1.80; p = 0.077)

### Genotyping

The *COMT *val158 met polymorphism rs4680 was genotyped by ABI TaqMan^® ^technology [38]. Several DNA replicates, reference DNA samples and negative controls without DNA were included to ensure the accuracy of the SNP genotyping assay. TaqMan^® ^probes and primers were obtained from the Assay-on-Demand genotyping product provided by Applied Biosystems (Applied Biosystems, Foster City, CA, USA). For each individual DNA sample, 6 ng of genomic DNA was amplified in a total volume of 5 μl containing both allele probes labeled with 5'-VIC or 5'-FAM fluorophore and 2.5 μl of TaqMan^® ^universal PCR master mix. Amplification reaction conditions were 10 min at 95°C, followed by 50 cycles of 95°C for 15 sec and 60°C for 1.5 min. Allelic discrimination analysis was performed on the Prism 7900HT Fast Real-Time PCR system using the software SDSv2.2.2 (Applied Biosystems, Foster City, CA, USA). Quality criteria of genotyping were as follows: Minor allele frequency 0.412, call rate 100%, test for deviation from Hardy Weinberg Equilibrium p = 0.707.

### Paradigm

Sixty laser stimuli were applied to the dorsum of the left hand using a Thulium: YAG laser (Baasel Lasertech) with a wavelength of 2000 nm as described previously by several groups including our own [33,39-41]. Stimuli were applied from a distance of 3 cm at a 90° angle. The site of the stimulation was manually moved after each trial to avoid tissue damage. Pulse duration was 1 ms, stimuli were spots 6 mm in diameter, stimulus intensity was 600 mJ. The interval between stimuli was pseudo-randomized between 8-12 seconds. Every third laser-stimulus in the sequence was skipped to allow the hemodynamic response return to baseline. At the end of the experiment subjects were asked to rate verbally the perceived sensation on a numerical rating scale ranging from 0 to 100 where 0 was "no pain" and 100 "pain as bad as it could be" [42]. Laser stimuli elicited a clear pinprick sensation in all 57 subjects. The *post-hoc *average pain rating was 42.1 (SD 15.6) points out of 100. Additional electrophysiological data (electroencephalography (EEG) and electrodermal activity (EDA)) were simultaneously obtained but were not considered for the present analysis.

### fMRI data acquisition

Functional MR-images were acquired using a 3T scanner (Trio, Siemens, Erlangen, Germany). In order to avoid head movements, the head of each subject was tightly fixated during the scanning procedure with vacuum cushions and sponge pads. Using echo planar imaging (EPI), 350 volumes were obtained applying the following EPI parameters: 44 slices, no gap, slice thickness 3 mm, FOV 192 × 192 mm, matrix 64 × 64, repetition time 2,670 ms, echo time 30 ms, flip angle 90°. To facilitate localization and co-registration of functional data, structural scans were acquired using T1-weighted MRI sequences (Magnetization prepared rapid gradient echo (MP-RAGE): TR/TE = 1,700/3.5 ms, flip angle = 9°, 208 sagittal slices, FOV 240 × 195 mm, matrix 320 × 260, voxel size 0.75 × 0.75 × 0.75 mm.

### fMRI analysis

fMRI-analysis was performed with FSL (FMRIB's Software Library, http://www.fmrib.ox.ac.uk/fsl). The following pre-processing procedure was applied: Employing different modules of the FSL-software package, we conducted motion correction using MCFLIRT [43], non-brain removal using BET [44], spatial smoothing using a Gaussian kernel of FWHM = 6 mm, mean-based intensity normalization of all volumes by the same factor, and highpass temporal filtering (sigma = 30 seconds). Whole brain general linear model (GLM) time-series statistical analysis of individual data sets was carried out using FILM (FMRIB's Improved Linear Model) with local autocorrelation correction [45]. Registration of functional images to high resolution structural images was done with FLIRT [43,46]. For the analysis of functional data, we used the time course of laser stimuli as the explanatory variable (EV) convolved with a Double-Gamma hemodynamic response function. The Double-Gamma function is a mixture of two Gamma functions - a standard positive function at normal lag (6 seconds) and a small delayed, inverted Gamma to model the late undershoot [47].

Group level mixed effect analyses were conducted using FLAME (FMRIB's Local Analysis of Mixed Effects) [48] with spatial normalization to MNI (Montreal Neurological Institute) space and applying a cluster significance threshold of Z > 2.3 [46,49,50]. Differences between genotype groups were investigated using two sample t-tests. For visual display of the group results, Z-maps of the functional data were imported to MRIcron [51].

### fMRI genotype effect analysis

**Figure 3:**
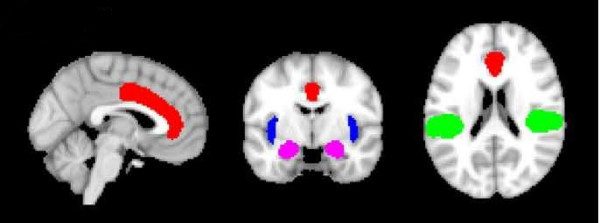
**Regions of interests**. Red - anterior cingulate cortex, blue - insula, green - parietal opercular cortex, purple = amygdalae.

Given the sample size of the present study, we primarily adopted a region-of-interest (ROI) approach for the analysis of genotype effects in order to reduce the degrees of freedom in the data space. For ROI analyses four anatomical masks were created (Fig. 3): a) bilateral parietal opercular cortex containing the secondary somatosensory cortex (S2) b) bilateral insula and c) bilateral anterior cingulate cortex (ACC), d) bilateral amygdala. These regions were chosen because they are known to be key areas of cortical pain processing, often referred to as the 'pain matrix' or important interconnected structures [5,6]. The masks were created using the Harvard Oxford atlas tool for cortical and subcortical structures which is implemented in the FSL software package. Within the resulting ROIs, the mean Z-value was calculated. These masks were also used for small-volume correction of group-level mixed-effects FLAME analysis of activation differences between *COMT *genotype groups. Here, differences between genotype groups were investigated using two sample t-tests.

### Statistical analysis

All statistical analyses were performed using the SPSS 15 software package (SPSS Inc. Chicago, Il, USA). Two-sample t-tests or chi-square tests were used as appropriate to determine the statistical significance of differences in demographic variables between genotype groups. The statistical significance of between-genotype group differences in fMRI region-of-interest activation measures was tested using a multivariate GLM analysis of variance (MANOVA) with *COMT *genotype as factor, and mean Z-values in the four ROIs as dependent variables. Subsequent "post-hoc" analyses of variance (ANOVAs) for each of the four ROIs separately were also performed.

## Competing interests

The authors declare that they have no competing interests.

## Authors' contributions

AM designed the study, analysed fMRI data and wrote the manuscript. JB performed experiments and was involved in data analysis. HT performed the genotyping, contributed to the preparation of the manuscript. MRT performed genotyping. MS was involved in genotyping, data base management and statistical data analysis. TW performed experiments and contributed to the preparation of the manuscript. FM performed experiments and contributed to the preparation of the manuscript. HW provided scanner hardware, was involved in preparation of the study and the manuscript. AS provided scanner hardware, was involved in preparation of the study and the manuscript. AS was involved in designing and preparing the study, provided hardware, contributed to the preparation of the manuscript. GW was involved in designing the study and preparing the manuscript. All authors read and approved the final manuscript.
